# Effects of a new outdoor mosquito control device, the mosquito landing box, on densities and survival of the malaria vector, *Anopheles arabiensis,* inside controlled semi-field settings

**DOI:** 10.1186/s12936-015-1013-8

**Published:** 2015-12-09

**Authors:** Arnold S. Mmbando, Fredros O. Okumu, Joseph P. Mgando, Robert D. Sumaye, Nancy S. Matowo, Edith Madumla, Emmanuel Kaindoa, Samson S. Kiware, Dickson W. Lwetoijera

**Affiliations:** Environmental Health and Ecological Sciences Thematic Group, Ifakara Health Institute, PO Box 53, Ifakara, Tanzania; Faculty of Health Sciences, School of Public Health, University of the Witwatersrand, Johannesburg, South Africa; Faculty of Health Sciences, School of Pathology, University of the Witwatersrand, Johannesburg, South Africa; Department of Mathematics, Statistics and Computer Science, Marquette University, Milwaukee, WI USA; Vector Biology Department, Liverpool School of Tropical Medicine, Pembroke Place, Liverpool, L3 5QA UK

**Keywords:** Mosquito landing box, Malaria, Elimination, *Anopheles arabiensis*, Pirimiphos methyl, Outdoor biting, Pyriproxyfen, *Metarhizium anisopliae*, Semi-field system

## Abstract

**Background:**

The significance of malaria transmission occurring outdoors has risen even in areas where indoor interventions such as long-lasting insecticidal nets and indoor residual spraying are common. The actual contamination rates and effectiveness of recently developed outdoor mosquito control device, the mosquito landing box (MLB), on densities and daily survival of host-seeking laboratory *Anopheles arabiensis*, which readily bites humans outdoors was demonstrated.

**Methods:**

Experiments were conducted in large semi-field systems (SFS) with human volunteers inside, to mimic natural ecosystems, and using MLBs baited with natural or synthetic human odours and carbon dioxide. The MLBs were dusted with 10 % pyriproxyfen (PPF) or entomopathogenic fungi (*Metarhizium anisopliae*) spores to mark mosquitoes physically contacting the devices. Each night, 400 laboratory-reared *An. arabiensis* females were released in one SFS chamber with two MLBs, and another chamber without MLBs (control). Mosquitoes were individually recaptured while attempting to bite volunteers inside SFS or by aspiration from SFS walls. Mosquitoes from chambers with PPF-treated MLBs and respective controls were individually dipped in water-filled cups containing ten conspecific third-instar larvae, whose subsequent development was monitored. Mosquitoes recaptured from chambers with fungi-treated MLBs were observed for fungal hyphal growth on their cadavers. Separately, effects on daily survival were determined by exposing *An. arabiensis* in chambers having MLBs treated with 5 % pirimiphos methyl compared to chambers without MLBs (control), after which the mosquitoes were recaptured and monitored individually until they died.

**Results:**

Up to 63 % (152/240) and 43 % (92/210) of mosquitoes recaptured inside treatment chambers were contaminated with pyriproxyfen and *M. anisopliae*, respectively, compared to 8 % (19/240) and 0 % (0/164) in controls. The mean number of larvae emerging from cups in which adults from chambers with PPF-treated MLBs were dipped was significantly lower [0.75 (0.50–1.01)], than in controls [28.79 (28.32–29.26)], P < 0.001). Daily survival of mosquitoes exposed to 5 % pirimiphos methyl was nearly two-fold lower than controls [hazard ratio (HR) = 1.748 (1.551–1.920), P < 0.001].

**Conclusion:**

High contamination rates in exposed mosquitoes even in presence of humans, demonstrates potential of MLBs for controlling outdoor-biting malaria vectors, either by reducing their survival or directly killing host-seeking mosquitoes. The MLBs also have potential for dispensing filial infanticides, such as PPF, which mosquitoes can transmit to their aquatic habitats for mosquito population control.

## Background

Controlling human-vector interactions has a central role in efforts towards malaria elimination by protecting humans from potentially infectious mosquito bites, reducing pathogen transmission [1–3]. Over the past decades, use of long-lasting, insecticide-treated nets (LLINs) and indoor residual spraying (IRS) against indoor-biting and indoor-resting malaria vectors has significantly lowered the burden of malaria transmission through personal and communal protection [4–8]. Despite these gains, there is ongoing transmission, a significant proportion of which now occurs outdoors and is not directly preventable using quality-assured indoor interventions such as LLINs or IRS [9]. Outdoor vector biting patterns are driven primarily by differential effects of indoor insecticidal interventions on mosquitoes that preferentially feed and rest indoors [10, 11], and human behaviours, such as people spending most time outside their houses in evenings performing different activities [12–14].

Despite the significance of outdoor malaria transmission as a challenge for malaria elimination efforts [15, 16], there are still no reliable and scalable tools developed to address this sub-set of transmission [17]. Fortunately, there are some definitions of what the target products should look like to effectively protect humans against malaria vectors while outdoors [18]. Amongst the candidate products that have been proposed and tested are: (1) spatial repellents which have proven effective for practical community uses either in vapour or aerosol formats [19–21]; (2) topical repellents such as DEET [22, 23]; (3) insecticidal treated clothing [24, 25]; (4) insecticide treated cattle [26, 27]; (5) larval source management [28, 29]; and, (6) toxic sugar baits [30, 31].

In addition, odour-baited devices have been proposed as potential complementary tools, which can be used alongside LLINs in Africa to disrupt malaria transmission. The recently developed Ifakara odour-baited stations (OBS) [32], and mosquito landing boxes (MLBs), which are baited with synthetic human odours [33] have been demonstrated to effectively attract, contaminate and/or kill host-seeking mosquitoes [34, 35]. The MLB has been described previously by Matowo et al. [34] as an outdoor vector control tool which mimics humans while outdoors. To contaminate and kill significant numbers of malaria vectors using MLBs, the device must be augmented with agents which either kill instantly, such as electrocuting grids [36], highly potent insecticides [32, 37], or those which kill/contaminate slowly at small doses, such as mosquito-killing fungi [35, 38] or pyriproxyfen, an insect juvenile hormone analogue, which also sterilizes insects [39–41].

An earlier study conducted under full field settings demonstrated that odour-baited MLBs attract significant numbers of both malaria and non-malaria vectors. However, the attracted mosquitoes were found to spend less time on the devices before they eventually flew away [34], presumably because the mosquitoes quickly realized that the device is a decoy (pseudo-host), with no vertebrate blood. However, this behaviour could also be due to behavioural avoidance to lethal contact as previously observed in malaria mosquitoes [42–45]. The initial study with MLBs conducted against natural populations of free-flying mosquitoes, could not quantify mosquitoes that made contact with these MLBs nightly because of lack of appropriate markers for field mosquito populations [3]. In addition, the actual impact of the devices on daily survival and densities of host-seeking mosquitoes was not established in the study. However, demonstrating full potential of vector control interventions [3], including odour-baited devices [46] require systematic quantification of these fundamental parameters.

The current study was conducted under controlled environments inside two large semi-field system (SFS) cages, i.e., large screen houses, available at Ifakara Health Institute (IHI), in southeastern Tanzania. The main aim was to evaluate actual effects of odour-baited MLBs on daily survival probabilities, and on human biting densities of the malaria vector, *Anopheles arabiensis,* which is today one of the predominant vector species in rural Africa, particularly in areas where LLINs and IRS have effectively controlled *Anopheles gambiae* s.s. [47–49]. To assess effects of MLBs on mosquito survival, the devices were treated with a paint-based mixture of pirimiphos methyl (actellic) as a candidate killing agent, whereas to assess its impact on vector densities, pyriproxyfen (PPF) or entomopathogenic fungi (*Metarhizium anisopliae* IP46) were used so that mosquitoes that made physical contact with the device could be easily and visually identified. This way, PPF and the fungus effectively marked the mosquitoes and facilitated reliable assessment of whether any recaptured mosquitoes had actually visited and made contact with the MLB inside the SFS.

## Methods

### Study area

**Fig. 1:**
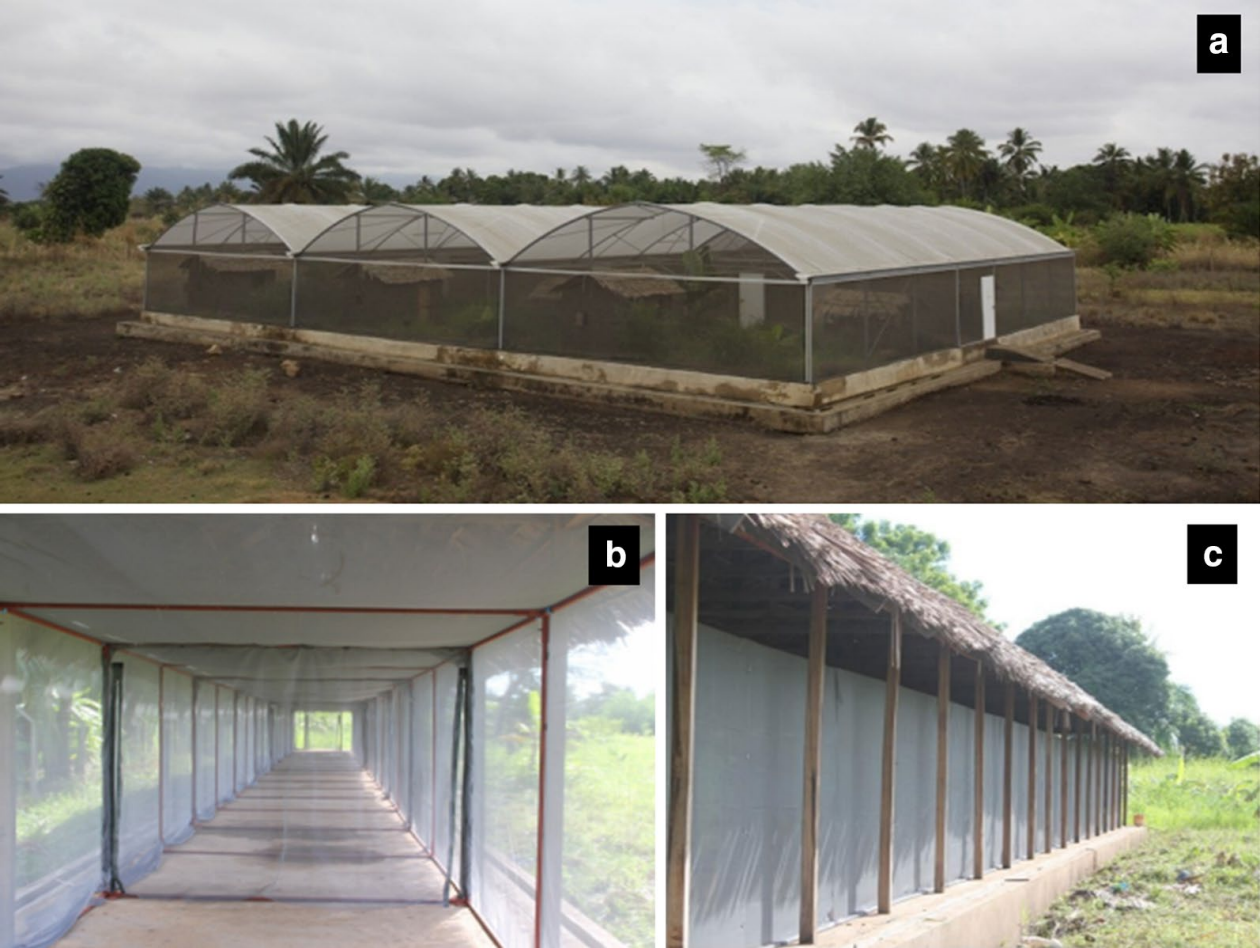
The semi-field systems used for the experiments. **a** shows the large multi-compartment system; **b** shows the inside sections of the long tunnel-shaped, semi-field system; **c** is an outside view of the long tunnel-shaped semi-field system

The study was conducted at IHI at Kining’ina experimental station (8.11417S, 36.67864E) in rural southeastern Tanzania. The tests were conducted inside two large screened cages, designed to create SFS. The first of these facilities (Fig. 1a) had six chambers each 9.6 m long × 9.6 m wide × 4.5 m high with 552.96 sq m total area, and was previously described in detail [40, 50, 51]. The second was a long screened-cage tunnel (Fig. 1B and C) measuring 120 m long × 2 m wide × 2.5 m high [19, 52]. During the experiments, average daily temperatures inside the SFS ranged between 20.5 and 29.0 °C, while relative humidity was between 50.5 and 80.0 %.

### Mosquito landing box

**Fig. 2:**
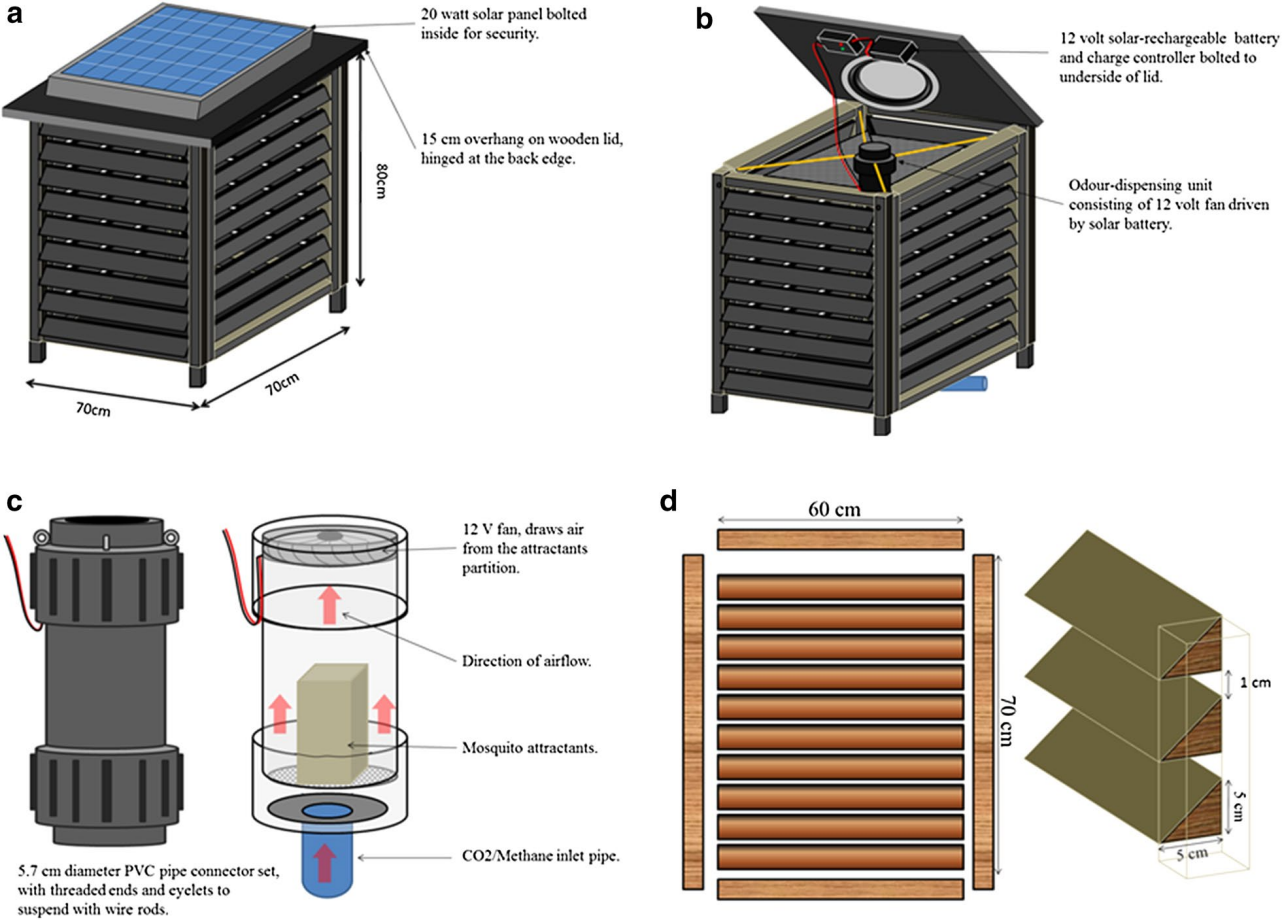
The odour-baited mosquito landing box. The MLB is designed to target mosquitoes, which bite humans while outdoors. It has a solar panel on *top* (**a**), which powers the fan on *top* of the odour-dispensing section inside (**b**, **c**). It is baited with natural or synthetic human odours, which attract malaria mosquitoes. Attracted mosquitoes are killed upon contact with chemical-treated louvres (**d**), or low-voltage electrocuting grids fitted on the inside sections behind the louvres and powered by the same solar panel [34]

Detailed information about the MLB has been provided by Matowo et al. [34]. In summary, it is a wooden solar-driven mosquito control box measuring 0.7 m × 0.7 m × 0.8 m, and standing on short wooden pedestals raised 10 cm above ground. It is designed to target host-seeking mosquitoes that bite outdoors at times when people are usually not protected by LLINs (Fig. 2a). It has side panels that are removable so that it is easy to transport and assemble onsite (Fig. 2d). Multiple louvres (8 or 12) are attached on each of the four side panels, which form the mosquito landing or mosquito contact surfaces. The louvres are 1 cm wide and are fixed at an angle of approximately 45° facing downwards, with gaps of about 2 cm between them (Fig. 2d) [34]. The MLB has a small odour-dispensing unit inside, where mosquito attractants are placed and dispensed with the aid of airflow from a 12-volt fan driven by a solar panel, and placed on top of the dispensing unit (Fig. 2b, c) [34]. Different numbers of louvres represent variation in surface area required for insecticides application. In this study, the MLBs with 12 louvres were used in all the experiments.

### Mosquitoes

All experiments were performed using insectary-reared *An. arabiensis* females aged 3–9 days post eclosion, from a colony originally from Lupiro village, in rural southeastern Tanzania, some 30 km south of Ifakara, and reared as previously described [53]. All mosquitoes were starved for 6 h before commencing the experiments each night to encourage host-seeking behaviour once the mosquitoes were released inside the SFS chambers. All the larval bioassays (as described below, to assess contamination status of the exposed adult mosquitoes) were performed using third-instar larvae of *An. arabiensis* from the same colony.

### Mosquito attractants

In the different experiments, the MLBs were baited with either human foot odour entrapped and preserved in nylon socks worn for 12 h before start of the experiments, or a blend of synthetic human odour previously developed at IHI [33]. In either case, the nylon socks or the synthetic attractant was supplemented with carbon dioxide (CO_2_) gas, produced from yeast-molasses fermentation [54, 55]. The yeast-molasses mixture was prepared at least one hour before commencing the experiments in two separate clean plastic pots, each containing 250 ml of molasses and 1.5 L water. The amount of yeast was different in each pot, with 40 and 75 g in the first and second pot, respectively. The yeast-molasses mixture was added in the pot in the order of yeast, molasses and, lastly, water, and then adequately stirred and left for at least 1 h before being placed inside the MLB.

### Pyriproxyfen, entomopathogenic fungi and pirimiphos methyl

The effects of treated the MLBs with PPF or entomopathogenic fungi on vector densities, were assessed at different experimental set-ups, so that to easily identify mosquitoes that made contact with the devices. PPF is an insect growth regulator [juvenile hormone analogue (JHA)], which mainly affects Diptera in their aquatic stages, such as larvae and pupae [56, 57]. It also affects the ability of adult mosquitoes to lay viable eggs and can sterilize male mosquitoes [41, 58, 59].

Entomopathogenic fungi are potent biological insect-killing agents, effective against a wide range of dipterans, including malaria vectors. They are capable of reducing daily survival of adult mosquitoes, since fungal conidia sporulate easily inside the mosquito bodies [35, 60], a characteristic which allows visual confirmation of fungal growth on the mosquito cadavers 8–9 days post exposure [61]. The strain, *M. anisopliae* IP 46, was originally isolated from soil from Brazil [35, 61, 62].

In this study, PPF and *M. anisopliae* were used as markers to assess whether host-seeking *An. arabiensis* had visited the MLB inside the SFS. In the experiments involving PPF, four sides of MLB louvres lined with black cotton cloth dampened with water were dusted with 3 g of 10 % active ingredient PPF powder (Sumilarv©, Sumitomo Chemical Co Ltd, Japan) per side (0.49 sq m) using a fine paint brush. The PPF-dusted MLB was left to dry for 8–10 h before commencing the experiment.

When entomopathogenic fungi were used in the experiments, a spore suspension of *M. anisopliae* was formulated by mixing the fungal spores with mineral oil [61, 63]. The suspension was sprayed on each MLB louvres on all four sides using a hand-held pressure sprayer (Minijet^®^, SATA, Germany) held 0.5 m away and perpendicular to the MLB to achieve a target concentration of 8 × 10^10^ spores/sq m.

The effects of MLB on vector survival, was assessed by treating the devices with a paint-based mixture of the toxicant, pirimiphos methyl (actellic) to demonstrate whether a treated odour-baited MLB can reduce daily survival rates of any mosquitoes that had made contact. Four sides of the selected MLB were painted using a 5 % pirimiphos methyl prepared using a 50 % emulsified concentration (EC) of pirimiphos methyl (Syngenta, Switzerland) mixed in oil paint. Pirimiphos methyl is a highly effective organophosphate recommended for IRS by WHO Pesticide Evaluation Scheme (WHOPES) [64, 65].

Using a hand-held fine paint brush, two MLBs were coated to achieve a thin uniform layer of the 5 % paint emulsion of pirimiphos methyl (i.e., 50 ml of the 50 % pirimiphos methyl emulsion mixed with 500 ml oil-based paint). The devices were kept in the shade for 48 h to dry before starting the experiments. UV-resistant netting material used to line the inside surfaces of the MLB were also treated, by soaking it in the 5 % pirimiphos methyl paint emulsion.

### Experimental design and procedures

#### Experiment 1: Demonstrating effects on malaria vector densities by assessing proportions of mosquitoes that are contaminated when MLBs treated with PPF are located inside the SFS

The first experiment was conducted inside the SFS, (Fig. 1a) for six nights, during which the PPF was used to contaminate mosquitoes inside the chamber. In the SFS treatment chamber two MLBs treated with PPF were placed 14 m diagonally apart, at opposite corners of the compartment. Two adult male volunteers sat inside the same SFS compartment, but at the remaining two opposite corners, and 14 m away from each other, performing human landing catches (HLC) [66].

A total of 400 female *An. arabiensis* mosquitoes were released each night at 18:30 h inside the chamber for six consecutive nights. Each night, starting 6 h after release of the mosquitoes, the volunteers started recapturing mosquitoes from within the SFS chambers by aspirating those mosquitoes that attempted to bite them on their limbs by using the HLC method. To avoid PPF cross-contamination between collected mosquitoes, each volunteer had 20 differently labelled aspirators and cups for aspirating and holding the recaptured mosquitoes individually. Each night, the volunteers were instructed to collect only 20 mosquitoes (ten collected while attempting to bite them on the legs and another ten collected from the netting walls and other surfaces within the SFS). The control setting in this experiment consisted of a similar setting in a different SFS chamber, but without the MLBs. Recapturing of mosquitoes in the control SFS chambers was conducted using the same protocol, except that each of the two volunteers used only one aspirator to collect all mosquitoes, as there was no risk of contamination in the control chamber.

The collected mosquitoes in both treated and control chambers during the first experiment were killed by refrigeration, and each mosquito was suspended in 50 ml of water containing ten third-instar larvae of laboratory-reared *An. arabiensis* to monitor larval mortality and pupa emergence inhibition. In addition, aspirators used to collect individual mosquitoes were rinsed with water to remove any possible PPF particles and clean water added so that the total was the 50 ml for each larval bioassay [40]. It was assumed that if an adult-recaptured mosquito was dipped in a cup with third-instar larvae, the larvae would die, confirming that the adults actually had made physical contact with the MLBs. On the other hand, if the larvae survived, then the dipped adult mosquito would be considered as not contaminated. In this case, physical contact with the device would not be confirmed.

During larval bioassay, each cup was monitored individually on a daily basis for larval mortality, pupation and adult emergence until all larvae died or until they had developed into pupae and emerged as adults. The emerged adults in each cup were removed, killed and recorded. The mean mortality observed in the cups was corrected using the Abbot’s formula [40, 67]. The proportion of recaptured adult female mosquitoes contaminated with PPF in the treatment chamber was calculated by setting a maximum mortality threshold at the upper 95 % confidence interval from the mean mortality in the control set-ups (i.e., minimum acceptable mortality beyond which effects of PPF must be attributed = mean mortality in controls + 1.96 SD). This way, a threshold mortality of 20 % was established. Any observed larval or pupal mortality in a bioassay cup above this set threshold of 0.2 from the treatment arm implied that the suspended mosquito was contaminated [40].

#### Experiment 2: Demonstrating effects on malaria vector densities by assessing proportions of mosquitoes that are contaminated when MLBs treated with *Metharizium anisopliae* are located inside the SFS

This experiment was conducted for 14 trap-nights to assess proportions of mosquitoes that visited and were contaminated by the MLB when placed inside the 120-m long semi-field tunnel (Fig. 1b) [19], in which human volunteers slept under exposure-free tents. Two odour-baited MLBs sprayed with fungal spores were introduced into the screened-cage tunnel [19]. In addition, two exposure-free Ifakara tent traps (ITTs) [68] with sleeping, male, adult volunteers were also set inside the SFS, so that the mosquitoes had a choice between actual human attractive cues and odours from the MLB. The MLB and the tent trap were 30 m apart. A total of 400 female *An. arabiensis* were released inside the tunnel each night for seven consecutive nights. Each morning the mosquitoes were recaptured inside the tent traps and from the general area within the tunnel, including walls and ceiling of the SFS. Plastic bowls with 10 % glucose solution were provided inside the tunnel, so that the remaining mosquitoes (not recaptured either in the tent traps or by aspiration) could survive on the sugar. Collected mosquitoes were monitored for fungal growth as previously described by Mnyone et al. [61, 69]. Visualization of hyphae growth on the mosquito cadavers, a proxy indicator for fungal infection, was used to assess the proportion of mosquitoes contaminated with entomopathogenic fungi [35, 70]. To avoid contamination between mosquitoes recaptured from the treated and untreated tunnel chambers, the control experiment was run first, and followed by the treatment using the same protocol. In the control setting, the MLBs were only treated with an oil formulation.

#### Experiment 3: Demonstrating effects on vector survival by assessing daily survival rates of mosquitoes exposed to MLBs treated with pirimiphos methyl organophosphates

**Fig. 3:**
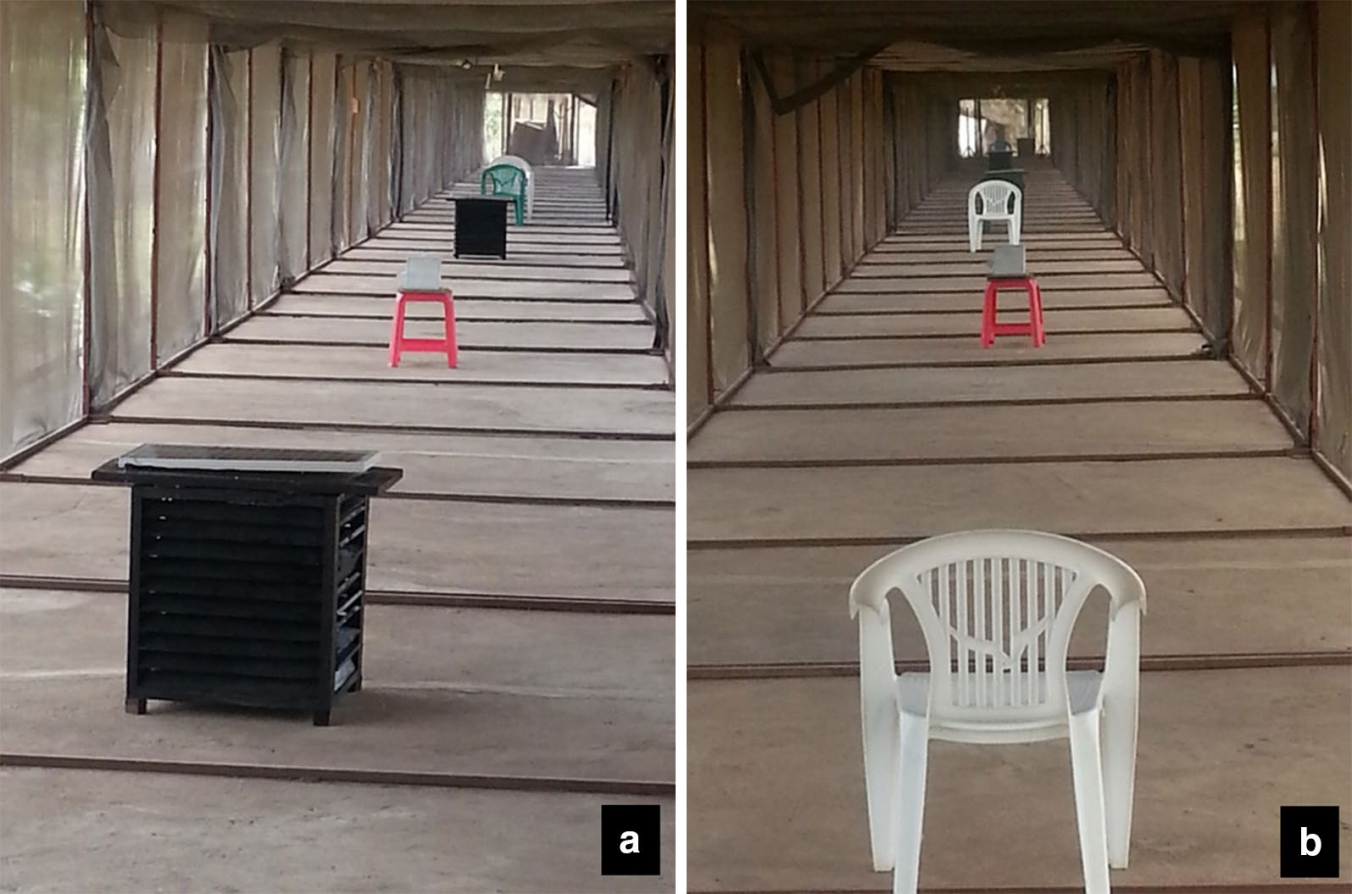
Pictorial representation of daily mean survival rate experiment set-up, treated chamber (with MLB treated with 5 % pirimiphos methyl mixed in oil based paints) (**a**), and control chamber (without the MLB), (**b**) inside mosquito tunnel

To assess daily survival rates of mosquitoes exposed on the odour-baited MLB painted with 5 % pirimiphos methyl emulsified paints [34], a study was conducted for three consecutive weeks (four nights per week, i.e., a total of 12 nights) inside the 120-m long tunnel (Fig. 3a, b). Two separate tunnel chambers measured 60 m × 2 m × 2.5 m [19] were used for treatment and control experiments. A total of 800 female *An. arabiensis* mosquitoes, 400 inside each SFS chamber, were released nightly at 18:30 h. Recapturing of the mosquitoes began 6 h afterwards so that these mosquitoes could acclimatize to the environment. A comparative binary crossover experimental design [71] was used. In the treatment chamber, two MLBs painted with the insecticide-based mixture were placed 40 m apart, alongside two adult male volunteers, each at 10 m from the MLB positions and 60 m from each other (Fig. 3a). Each night, the volunteers entered the chamber 6 h after the mosquitoes had been released, instructed to recapture the first 20 mosquitoes attempting to bite them. In the control segment, no MLB were introduced, but two male volunteers stationed 30 m apart worked to recapture any mosquitoes attempting to bite them. In the treatment section, each volunteer had 20 different labelled aspirators, as well as paper cups for aspirating and holding mosquitoes individually, to avoid any pirimiphos methyl cross-contamination between collected mosquitoes. The control was concurrently performed using the same procedure, but without the MLB devices (Fig. 3b). Two volunteers collected mosquitoes inside the chamber, each using only one aspirator to collect all mosquitoes. This experiment was repeated but with volunteers beginning collections 12 h after the mosquitoes were released. All the collected mosquitoes were maintained on 10 % glucose solution, and their daily survival rate was monitored at an interval of 24 h, noting whether the individual mosquitoes were alive or dead.

### Data analysis

#### Differences between proportions of mosquitoes contaminated in control and treatment chambers

To determine the differences in proportion of recaptured adult mosquitoes that had been contaminated in the SFS in which MLBs treated with PPF had been placed, relative to the control, the percentage emergence and percentage mortality of the larvae introduced in the water contaminated by these recaptured adults were considered. First, the proportion of adult mosquitoes contaminated with PPF was calculated by setting a maximum mortality threshold above an upper 95 % confidence interval from mortality observed in a control section and further corrected using the Abbot formula [40, 67]. This way, a threshold mortality of 20 % was established. Any observed larval or pupal mortality probability in a bioassay cup above this set threshold from the treatment arm implied that the adult mosquito dipped inside the larval cup had been contaminated with PPF.

A generalized linear mixed model (GLMM) with binomial distribution for proportional data was then performed in R software V2.12.2 [72], using the lme4 package [73] for GLMMs. The treatment groups (i.e., control or treatment) were classified as a fixed effect while holding cups, physical position of mosquito collection, and replicate code were included in the mixed effects model as random effects. To obtain the best model, the full nested model (including all parameters as described in the experiment) was compared with a reduced model (i.e., trying different combinations with one parameter removed at each time). At each stage, the better model was selected by visually inspecting the resulting diagnostic plots of residuals against model fitted values.

Visualization of hyphae growth on the mosquito cadavers, a proxy indicator for fungal infection was used to assess the proportion of mosquitoes contaminated with entomopathogenic fungi [35, 70].

#### Differences in survival of mosquitoes retrieved from control and treatment chambers

Daily survival data were analysed in SPSS version 21 [74], using a Cox proportional hazard model [75, 76] to assess how daily survival probabilities of individual mosquitoes varied between days after treatment. Statistically significant differences in overall mortalities and daily mean survival probabilities in the form of hazard ratios (HR) were generated by comparing the survival curves of mosquitoes in the control and treatment groups, which indicated the relative daily risk of dying among mosquitoes collected from a treatment chamber relative to those collected from a control chamber [76, 77]. HR value of 1 indicated equal mortality rates between treatment and control, HR values >1 indicated significantly greater mortality rates in treatment than control and HR <1 indicated significantly lower overall mortality rates in treatment than control. Daily mean survival rate HRs were calculated and compared between control and treatment groups.

### Ethics

Before starting the study, volunteers were provided with explanations of the aims and the potential risks and benefits, after which written informed consents were obtained from them. To minimize risks of mosquito bites, the volunteers were provided with long-sleeved clothing with ventilated hoods and gloves during mosquito collections. All the participants were offered free malaria tests by RDT every 2 weeks and had access to treatment using the first-line drug, artemether-lumefantrine (Coartem^®^), if they became unwell, although no volunteer became unwell during these experiments. There was no prophylaxis given to volunteers because mosquitoes used were laboratory-reared females with no prior blood meals. Ethical review and approval was provided by the institutional review board of Ifakara Health Institute (Ref: IHI/IRB/NO.030) and the Medical Research Coordinating Committee at the National Institute of Medical Research in Tanzania (Ref: NIMR/HQ/R.8a/Vol. IX/1222). This paper got permission to publish from NIMR (NIMR/HQ/P.12 VOL XVII/25).

## Results

### Effects of the MLB on mosquito densities in tests using PPF

**Fig. 4:**
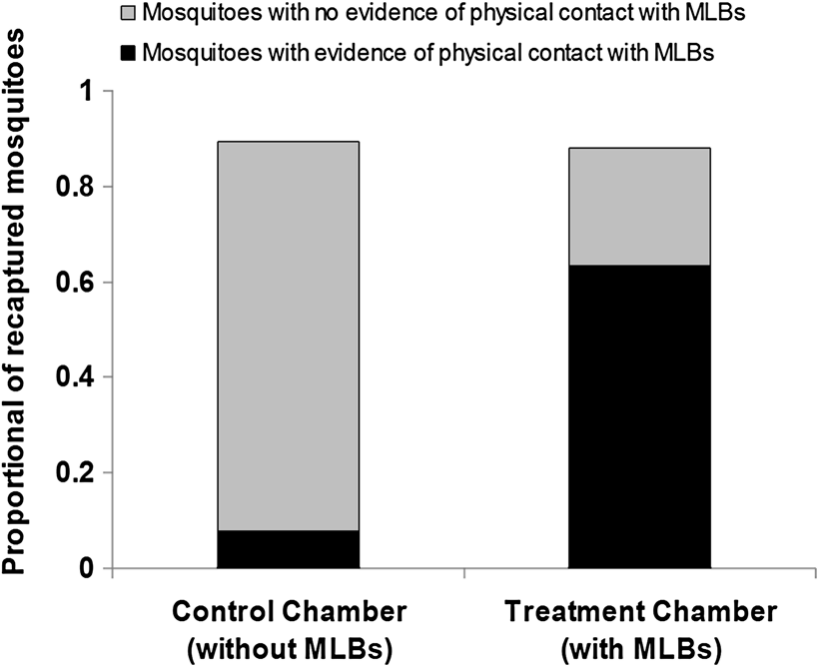
Proportion of adult recaptured *Anopheles arabiensis,* collected from control and treatment chambers that had evidence of having been contaminated with PPF. The proportion of mosquitoes contaminated per cup was determined based on the threshold limit of 20 % average emergence inhibition (EI) per cup (this being the estimated maximum possible mortality threshold above an upper 95 % confidence interval from mortality observed in a control). All recaptured mosquitoes resulting in ≥20 % EI of larvae were considered contaminated, or else uncontaminated

The proportions of exposed recaptured mosquitoes that made direct contact with the MLBs, were used as an indicator of how the devices would affect densities of malaria mosquitoes in any population. When the recaptured mosquitoes (which presumably had PPF on their bodies) were individually dipped into cups containing ten larvae (third-instars) in 50 ml of water, the recaptured mosquito was considered to have been contaminated (and therefore to have made direct physical contact with the treated MLBs), if >20 % of the larvae in the cups failed to emerge. Overall, there was a marked difference in average proportion of larvae emerging into adults when the cups were contaminated by exposed adults recaptured from the treatment relative to the control chambers of the SFS. The mean emergence rate in a control chamber was 28.79 [28.32–29.26], significantly higher than the mean emergence rate in a treatment chamber, which was 0.75 [0.50–1.01], (*P* < 0.0001). These differences existed regardless of whether the exposed adult malaria mosquitoes had been recaptured while attempting to bite the volunteer legs, i.e., via the HLC method or from the other surfaces within the SFS. There was no difference in exposure rates between mosquitoes recaptured on the volunteers relative to those recaptured on walls and other surfaces within the SFS (P > 0.05). In the treated chamber (i.e., chamber with MLBs treated with PPF) 63 % (152/240) were contaminated. In the control chamber (without MLBs), there was 8 % (19/240) of the recaptured mosquitoes which were associated with larval mortality during larval bioassay (Fig. 4).

### Effects of the MLB on mosquito densities in tests using entomopathogenic fungi, *Metharizium anisopliae*

When fungal spores were used as the marker on the surfaces of the MLB, which was located inside a SFS chamber with two adult human volunteers sleeping inside exposure-free tent traps, 43 % (92/210) of mosquitoes recovered inside the tents traps and 26 % (55/210) from SFS walls of the treatment chamber were found contaminated with *M. anisopliae* IP46, compared to 0 % (0/164) in the control chamber. It was also observed in the separate study that the mean survival time of the wild adult *An. arabiensis* after exposure to the *M. anisopliae* IP46 in the OBS was reduced five-fold, i.e., from 10.0 (2.8–14.3) days to 2.0 (1.0–4.0) days, HR = 2.65, P < 0.001 [35].

### Effects of the MLB on mosquito daily survival rates in tests using organophosphate, pirimiphos methyl

**Fig. 5:**
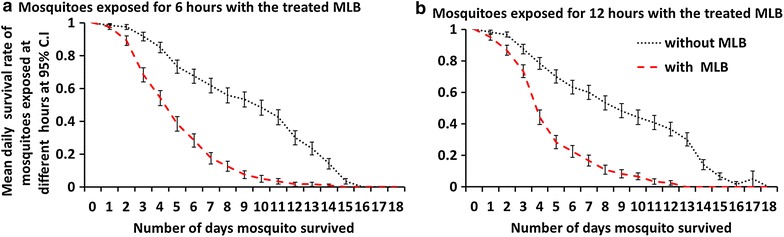
Daily mean survival rate of adult *Anopheles arabiensis* exposed in either control chambers or chambers with MLBs painted with the mixture of emulsified paints and 5 % pirimiphos methyl organophosphates. **a** shows results where mosquitoes were exposed for 6 h before recapturing began, while **b** shows results of experiments where the mosquitoes were exposed for 12 h before recapturing began

There was significance difference in survival rate between the control and the treatment P < 0.005. The probability of survival for the recaptured mosquitoes within the control group was 1.748 (1.551–1.920), higher than the treatment (P < 0.005). There were no statistical differences in survival for mosquitoes recaptured at different exposure times, 6 or 12 h (P > 0.005). However, at day 8, daily mean survival rate of all exposed mosquitoes in a treatment was 7.5 times lower than control group (Fig. 5). The experimental results indicate that an MLB, when treated with lethal insecticides such as pirimiphos methyl, can help reduce the daily survival rate of outdoor host-seeking mosquitoes 2 days every 24 h, compared to a control with no MLB.

## Discussion

Odour-baited technology has been proposed as potential new tools, not only for sampling, but also for controlling mosquitoes outside people’s houses [33, 46]. This study was conducted under controlled conditions inside large semi-field settings to demonstrate possible potential effectiveness of a recently developed outdoor mosquito control device, the MLB [34], on female adult malaria vectors. The MLB and its predecessor prototypes were designed to be used with a variety of mosquito-killing agents, including biological agents such as entomopathogenic fungi [35, 52], JHA [40, 59], toxicant insecticides [34, 52], and even electrocuting grids [36, 78]. In this study, PPF powder and fungal spores were selectively used as candidate contaminants that also offered capability to visually mark mosquitoes that made contact with the devices. This enabled estimation of proportions and densities of mosquitoes that could be affected whenever the device was used. In addition, pirimiphos methyl organophosphate was used as a candidate-killing agent, to demonstrate the effects of the device on the mean daily survival of any mosquitoes making physical contact. Using this approach, this study has clearly demonstrated high efficacy of the odour-baited MLB on mean daily survival rates but also the contamination success rate of laboratory *An. arabiensis* and possible impact on population densities reduction.

The observations of fungal hyphae on mosquitoes recaptured from within the SFS even in the presence of human hosts not only indicate potential benefits of the device for targeting malaria vectors, but also demonstrate that this device can be effective even where there are competing human hosts in the environment. It has potential for controlling outdoor-biting malaria mosquitoes in communities where people spend significant time performing various outdoor activities [14]. Although the efficacy of MLBs in attracting wild malaria vectors has been demonstrated previously [34], use of entomopathogenic fungal spores as a visual marker ensures easy and clear visualization of mosquito numbers making contact with the device [35], due to the ability of fungal spores to grow inside contaminated mosquitoes, even if only a few spores are acquired [60, 79, 80]. This marking technique is sensitive in detecting and estimating actual numbers of mosquitoes making very short contact with the MLB. In the experiment with fungal pathogens, the human volunteers slept inside exposure-free tent traps [81], which made possible to retrieve and segregate mosquitoes as those actively seeking humans *versus* those flying around but not necessarily host seeking. The confirmation of fungal infections on the mosquitoes collected inside the tent traps indicates that mosquitoes were at first attracted towards the MLB before searching for a real human. This observation emphasizes efficacy of the device even in the presence of humans. Moreover, the attractiveness of the MLB as a competitive pseudo-host over the human can be improved by varying the baits (for example, by using the highly attractive odour-baits or by varying distances and positioning of the devices within the target environments [33, 46].

Similarly, by using PPF as a candidate contaminant and marker, this study shows that the MLB has the potential of contaminating significant proportions of wild mosquitoes, in this case an average of 55 % of all mosquitoes exposed each night relative to the control. The recorded larvae mortality as the result of contaminated (8 %) total mosquitoes collected was not expected, and it was interpreted as natural larval mortality or cross-contamination during mosquito processing for larval bioassay. While this outcome may be reduced in field settings where malaria mosquitoes are free-ranging, as opposed to using captive insects as in experiments of this study, such a challenge can be easily countered by using highly attractive baits with long-range capability to attract mosquitoes from far and concentrate them in a small area. Previous studies have shown that attractants such as the Ifakara blend of synthetic odours work as medium- to long-range rather than short-range attractants that were used in this study [33]. Therefore, it could be used to ensure higher contact rates even in field settings, possibly achieving equal or higher contamination rates than observed in this study.

One limitation of this study was that same number of mosquitoes was released within the SFS daily. It was therefore not possible to test how the device would perform under conditions of varying vector densities.

Where PPF is used as the selected treatment for these devices, it would be possible to achieve a multiplicative effect, whereby the contaminated mosquitoes would also transfer the particles to their breeding sites and contaminate their progeny but also sterilize themselves, as previously demonstrated [40, 59]. In this study, emergence inhibition rates as high as 75 %, when exposed female *An. arabiensis* mosquitoes where dipped in larval bioassay cups containing thid-instar larvae of the same species was observed. It should also be noted that, whereas in nature mosquitoes could have multiple contacts with the devices, the PPF experiment allowed only six and later 12 h of exposure before the volunteers moved into start recapturing the mosquitoes. Therefore, it was speculated that the mosquitoes that apparently were not contaminated (37 %) either did not visit the device or lost some of the PPF in the act of resting and searching for human volunteers, prior to being recaptured [82]. The importance of PPF as a JHA and in the killing of aquatic stages of malaria vectors, mainly as pupae, has been documented recently [40, 83]. Although not investigated in this study, MLBs augmented with this compound can reduce the density of malaria vectors by causing sterility (reduced or/and blocked fecundity and fertility) of the contaminated mosquitoes [41, 59, 83, 84].

In assessing the efficacy of odour-baited MLBs treated with insecticides in killing and reducing daily survival rates of exposed *An. arabiensis*, it was observed that overall, the average risk of dying was approximately two times higher each day in the exposed group compared to a control. However, at day 8, the mean daily survival of all exposed mosquitoes in a treatment was 7.5 times lower than control group. The significantly elevated levels of mortality and reduction in mean daily survival at day 8 are particularly crucial for successful interruption of malaria transmission, as this ensures that the treatment can effectively block parasite (*Plasmodium* sp.) development inside the mosquito, which takes a minimum of 8 days to become infective in sub-tropical and tropical regions. Surprisingly, it was also observed that mean daily survival of mosquitoes was not affected by differences in time of exposure (Fig. 5). While the reasons for this observation was not investigated, it is likely that either the mosquitoes, after contacting the device once, did not revisit because the device did not offer a blood meal, or that the mosquitoes did not spend sufficient contact time with the device regardless of the duration of time the mosquitoes were inside the SFS alongside the device. This second hypothesis would mean that there were no differences in exposure time of mosquitoes to insecticide on the device, as previously suggested in the original description of the MLB [34].

Despite promising results, these findings should be interpreted cautiously since any development of new tools that rely on chemical insecticides could be affected by development of physiological or behavioural resistance in malaria vector populations, thus reducing efficacy under field settings. Practical use of MLBs in controlling outdoor malaria vectors will require it to be augmented with agents capable of killing mosquitoes at point of contact with the device, such as electrocuting grids [85], which have also been tried recently in Tanzania [36], chemicals which are potent in tiny dosages such as PPF [41, 84] or biological agents such as entomopathogenic fungi [60]. The efficacy of the killing agents would need to be assessed locally to ensure that the devices remain effective against vector populations. Nevertheless, overall, these results suggest that MLBs, if treated with effective killing agents, could have significant impact on densities and survival of outdoor-biting malaria mosquitoes, including *An. arabiensis,* which is increasingly becoming one of the dominant species in rural Africa following reduced occurrence of *An. gambiae* s.s. [47–49]. Given the importance of vector survival as a factor affecting malaria transmission [3], such an approach would likely be highly effective for the control of residual malaria transmission in Africa.

Based on the current and future challenges of using insecticide-based control interventions, observed differences in mosquito behaviours, and the need for a user-friendly, fast-acting device, a new automated MLB, fitted with low-cost, solar-driven, electrocuting grids has recently been tested with free-flying wild mosquitoes with promising results [36]. It is envisaged that the newly improved MLB could offer an extra killing mechanism against outdoor-biting mosquitoes and disease transmission occurring outdoors while complementing existing indoor control interventions such as LLINs. In addition to attracting and killing mosquitoes, this solar-driven device has a potential for supplying basic lighting to nearby households, thus improving community acceptability of the control intervention. Recent studies have demonstrated that such devices could be used to effectively dispense spatial repellents for protection of people sitting outdoors [86], thus increasing their range of usefulness.

To optimize the device, further evaluation will be necessary on how mosquitoes interact with the device, preferably by way of night-time, near-infrared and camera technology. Moreover, to achieve maximum impact during use, the devices should be preferentially located in places with high biting or high transmission risk. In recent analysis of vector-biting patterns in southeastern Tanzania [87] it was demonstrated that such high-risk locations can be identified readily based on easily available data, such as household occupancy. The efficacy of MLB, as described in this study, can be greatly enhanced by locating and targeting the high transmission pockets of malaria transmission [88].

## Conclusion

It was concluded that the MLB, when baited with effective mosquito attractants and augmented with effective mosquito-killing agents, can effectively attract, contaminate and kill outdoor host-seeking mosquitoes even in the presence of real human hosts. The devices could reduce mean daily survival rates and/or biting densities of adult malaria vectors significantly, and possibly reduce associated malaria transmission when used consistently. This study revealed the potential of the MLB as a way to dispense novel mosquito control compounds such as PPF, which mosquitoes can pick up and transfer to their own breeding sites, and is proven to effectively disrupt development of the aquatic stages of the mosquito progeny, and also sterilize the adult mosquitoes. To ensure that the device could be an effective complimentary strategy to be used alongside LLINs and IRS, further testing of the odour-baited MLBs in controlling outdoor-biting malaria vectors, to establish their entomological and epidemiological impacts in village settings, and also to estimate the associated costs when scaled-up are recommended.
